# Veins Improve Fracture Toughness of Insect Wings

**DOI:** 10.1371/journal.pone.0043411

**Published:** 2012-08-22

**Authors:** Jan-Henning Dirks, David Taylor

**Affiliations:** Department of Mechanical and Manufacturing Engineering, Trinity Centre for Bioengineering, Trinity College, Dublin, Ireland; Massachusetts Institute of Technology, United States of America

## Abstract

During the lifetime of a flying insect, its wings are subjected to mechanical forces and deformations for millions of cycles. Defects in the micrometre thin membranes or veins may reduce the insect’s flight performance. How do insects prevent crack related material failure in their wings and what role does the characteristic vein pattern play? Fracture toughness is a parameter, which characterises a material’s resistance to crack propagation. Our results show that, compared to other body parts, the hind wing membrane of the migratory locust *S. gregaria* itself is not exceptionally tough (1.04±0.25 MPa√m). However, the cross veins increase the wing’s toughness by 50% by acting as barriers to crack propagation. Using fracture mechanics, we show that the morphological spacing of most wing veins matches the critical crack length of the material (1132 µm). This finding directly demonstrates how the biomechanical properties and the morphology of locust wings are functionally correlated in locusts, providing a mechanically ‘optimal’ solution with high toughness and low weight. The vein pattern found in insect wings thus might inspire the design of more durable and lightweight artificial ‘venous’ wings for micro-air-vehicles. Using the vein spacing as indicator, our approach might also provide a basis to estimate the wing properties of endangered or extinct insect species.

## Introduction

During the lifetime of an insect, many parts of its exoskeleton are subject to external stresses and mechanical impacts, eventually causing small cracks by fatigue, wear and tear. If not repaired or at least stopped, cracks can eventually grow bigger, ultimately reducing the exoskeleton’s biomechanical function and as a consequence the insect’s fitness.

One part of the insect body which needs to resist repeated high mechanical stresses are the wings; in particular those of long-distance flying insects such as the desert locust *Schistocerca gregaria.* In their migratory stage, these insects can fly for days over several thousand kilometres in search of new habitats [1]. During this time their wings are subject to deformation, torsion and bending for millions of cycles. How do locust hind wings cope with damage, often resulting from interactions (antagonistic or sexual), collisions or fatigue [1]–[3]?

In most biological materials small defects due to wear and tear are inevitable and, rather than trying to prevent cracks, many organisms have adapted to either repair or withstand small defects in their structural tissues, such as plant stems, bone and skin [4]–[6]. However, due to the histological structure and morphogenetic development of the wing membrane as part of the locust’s exoskeleton, the repair of cracks is not possible [7]. This leaves only the option to minimize the effect of small defects by stopping them as soon as possible. How do locust wings prevent small defects from growing into large cracks?

So far the only indication of a crack inhibiting morphological adaptation in locust wings has been proposed by Wootton *et al.* [3]. A small crimped band around the edge of the wing could help to distribute stress, thus possibly preventing tearing of the membrane [8]. However, this mechanism would only reduce the effect of tearing from the edge, and would be of no avail for defects starting within the wing. To prevent cracks from growing inside the wing, one can thus imagine two possible toughening mechanisms: i) a very tough membrane materials and/or ii) the wing veins as crack-inhibiting barriers.

### Properties of the Wing Membrane

A very tough wing membrane cuticle could minimize the risk of cracks developing in the first place and inhibit their propagation through the material. Recently we were able to show that the toughness of the locust hind leg cuticle is amongst the highest of any biological composite material, minimizing the risk of cracks developing during the jump. Is the cuticle of the locust wing membrane particularly tough, too?

In a previous study Wootton *et al.* have characterised the histology, morphology and stiffness of the *S. gregaria* wing membrane in great detail [3], [8], [9]. It is extremely thin (1.7 to 3.7 µm) and in the main consists of epicuticle only. This cuticle contains amorphous cross-linked proteins, no traceable amounts of chitin and only very little water. Tensile experiments with isolated sections of the locust wing membrane showed a mean isotropic stiffness of 9.89±3.47 GPa for the remigium and 3.70±2.71 GPa for the anal fan. However, there was no clear pattern in the distribution of stiffness along the wing, which lead subsequent studies to simplify the properties of the membrane and veins when modelling the wing [9]. It was also shown that in respect to its biomechanical function, the locust wing membrane acts as a “stressed skin”, with the cells playing an important role in combining structural stiffness with flexibility during the wing movement. Nothing is known about the fracture toughness or strength of the locust wing membrane, or of any other insect wing membrane.

### Properties of the Veins

**Figure 1:**
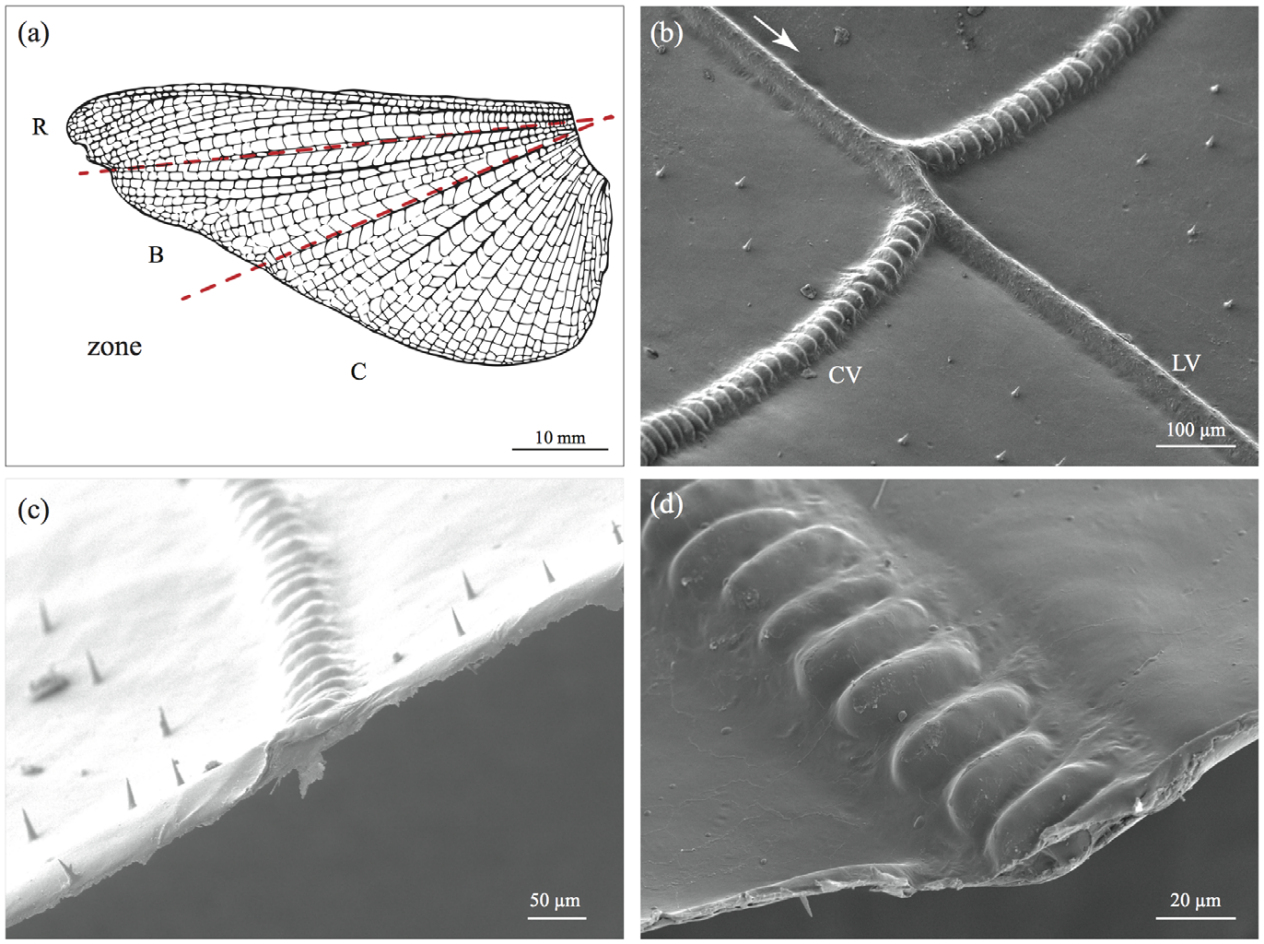
Morphology *of S. gregaria* hind wings. (a) Schematic illustration of the three wing zones R, B and C used for the experiments (adapted from [8]). (b) Longitudinal veins (LV) with branching cross veins (CV). Towards the edge of the hind wing the two types of veins show a different morphological structure. Whilst the longitudinal veins mostly show a circular to elliptical cross section, the cross veins show an annulated pattern. (c) Cross section through the wing membrane and a cross-vein. Note that the cutting edge of the wing membrane slightly “crumpled” during the desiccation. (d) Close-up of a cross-vein, showing the compartment-like annulated structure.

**Figure 2:**
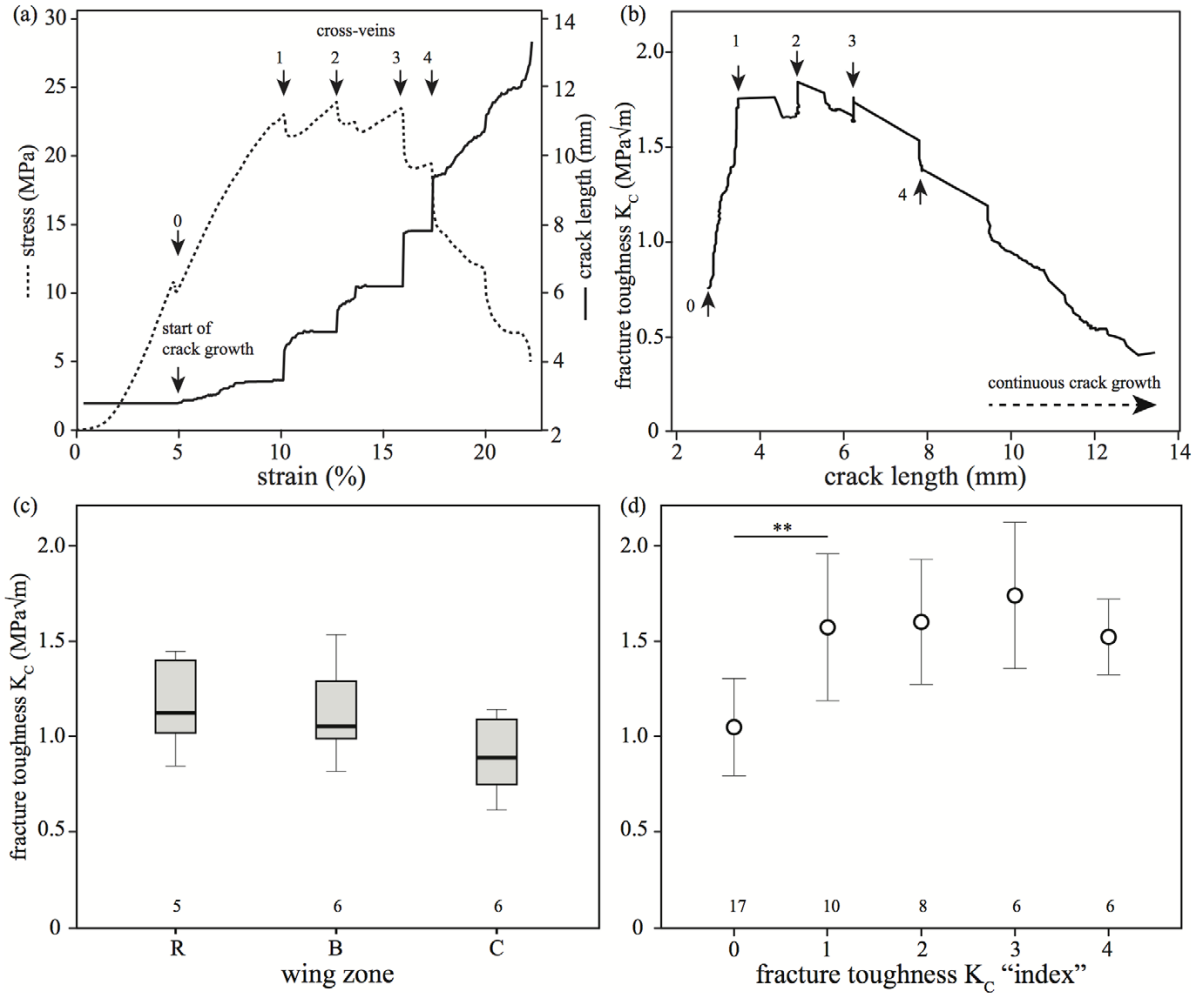
Crack propagation and fracture toughness of hind wings. (a) Stress-strain curve and corresponding crack length from one wing sample with an induced notch. The numbers indicate the K_C_ indices (see text). With increasing strain the stress on the wing membrane increases until the crack starts growing (0). When reaching cross veins (1–4), the crack propagation temporarily stops and the stress further increases. When the cross veins break, the stress decreases and the crack continues to propagate. (b) Crack length and corresponding fracture toughness K_C_. The markers 0–4 correspond to the markers in (a). (c) Fracture toughness of hind wing membrane. Although slightly decreasing towards the anal part of the wing, there was no significant difference in-between the fracture toughness K_C0_ of the membrane from the tested three wing zones (F_2,16_ = 2.087, p>0.1, ANOVA). (d) The membrane alone had a mean fracture toughness of 1.04±0.25 MPa√m (N = 17). The presence of the first cross-vein (index 1) significantly increased the fracture toughness of the wing structure to 1.57±0.38 MPa√m (t_9_ = −3.513, p<0.01, paired t-test, both figures show mean±SD, numbers show sample size).

**Figure 3:**
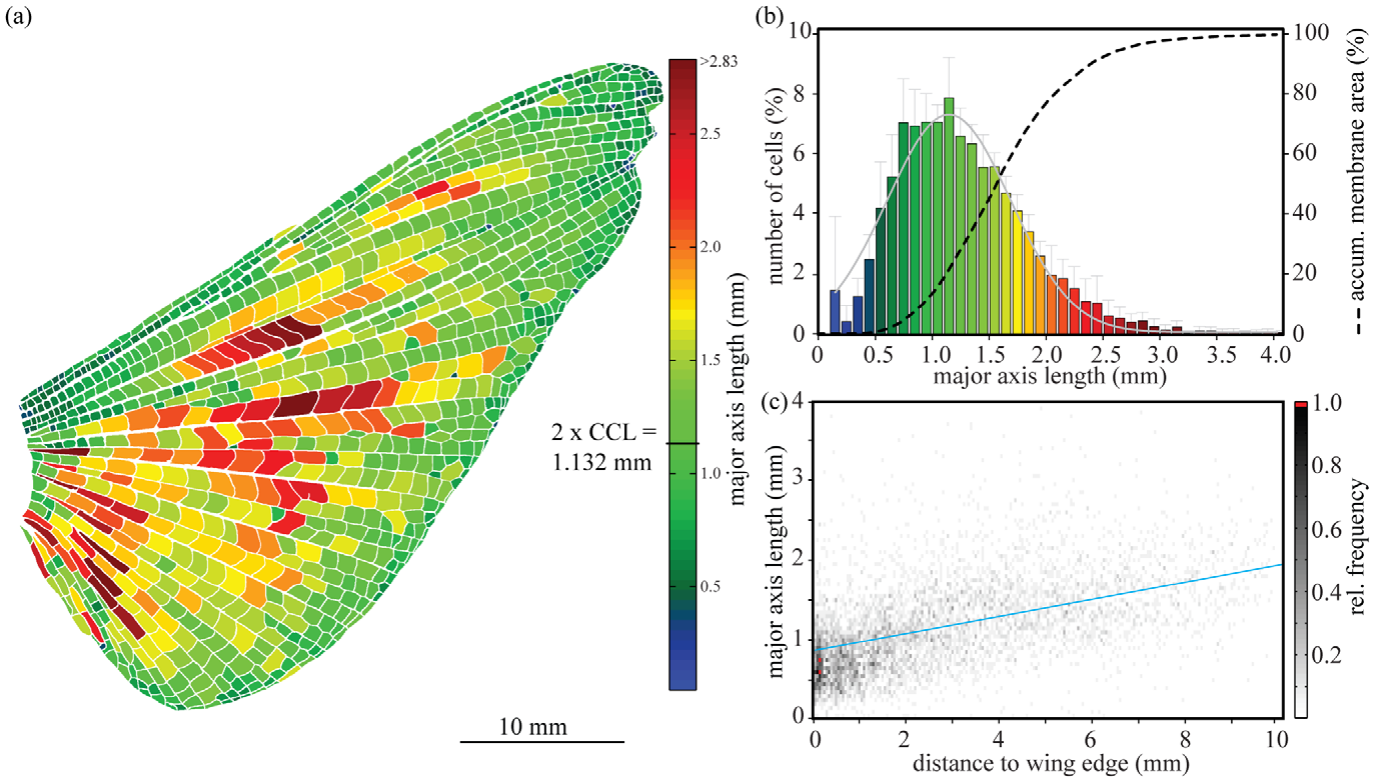
Size and distribution of wing cells in *S. gregaria* hind wings. (a) Typical structure of a hind wing, showing the distribution of the wing cells’ major axis length. Cells with smaller major axis lengths are mostly arranged around the perimeter of the wing (CCL: critical crack length). (b) Mean frequency of wing cell sizes from six hind wings. The distribution of cells corresponds very well to a normal distribution around a mean major axis length of 1.103 mm (σ = 544.16, a = 7.33, R = 0.98). The cumulative membrane area formed by cells smaller than the critical crack length is 19.44% of the overall membrane area (mean ± SD, N = 5553 cells from 6 wings). The colour map of the bars corresponds to subfigure A. (c) 2D-Histogram showing the relative frequency of cell size and their distance to the wing edge. There is a significant positive correlation of the major axis length with the distance to the wing edge (ρ = 0.393, R^2^ = 0.154, p<0.001, linear correlation, N = 5553 cells).

A network of longitudinal veins and cross-sectional veins divides the wing-surface into characteristic numerous smaller membrane cells (see Figure 1 and [10]). In locusts, the longitudinal veins are hollow cuticular tubes with a diameter of approx. 100 to 150 µm at the base, thinning towards the edge of the wing [9]. Most of the longitudinal veins contain trachea, nerves and hemolymph [2]. They branch distally and their beam-like structure provides stiffness and rigidity across the span of the wing and increases the resistance to torsion [9]. In a previous study it was shown that the arrangement of the longitudinal veins within the anal fan very closely follows a truncated logarithmic spiral around the base of the wing [11]. Using finite element modelling, it has been shown that this arrangement allows the hind wing to be mechanically unfolded like an umbrella during the downstroke [9]. The cross-veins however have a relatively diverse shape and contain little or no hemolymph [12]. In locusts, the typical annulated structure of the cross-veins is thought to increase the compliance of the cross-vein when in flexion (see Figure 1 and [2], [3]). Their role during insect flight has been far less studied than that of the longitudinal veins, however one of their main functions is believed to be to constrain the lateral buckling of the longitudinal veins and support the wing camber [11], [13]. There is no experimental data on the material stiffness, strength or fracture toughness of any of the veins [1], [9].

The aims of this study were to measure the fracture toughness of wing material and to investigate the role of the veins in preventing crack propagation.

## Methods

### Insects and Sample Preparation

Adult female *Schistocerca gregaria* desert locusts (bodyweight 2.022±0.46 g, N = 11) were taken from laboratory colonies kept at 12 h daylight/12 h night and fed with fresh vegetables and dried cereals *ad libitum*.

Hind wings were cut off as close to the wing base as possible. Previous experiments by Smith *et al.* have shown that, depending on the position on the wing, the membrane’s stiffness can vary [1]–[3], [8]. To test whether this was the case for toughness, too, we selected wing samples taken from three different zones of the wing following the ‘zoning’ by Wootton *et al*. [3]: zone ‘R’ including the remigium down to approx. the first anal vein 1A, zone ‘B’ starting at the 1A vein down to approx. the third anal vein 3A and zone ‘C’ from approx. the 3A vein on (see Figure 1 a).

During the downstroke, the umbrella-like unfolding of the hind wing leads to tensile forces perpendicular to the longitudinal veins [11]. To simulate these physiological stresses during our tests, the longitudinal veins were orientated parallel to the sample holders. For the mechanical tests fresh sections of the wings were immediately glued into small aluminium foil frames (approx. 10 mm width) using small amounts of fast hardening epoxy resin. Once the samples were mounted into the tensile grips, the supporting aluminium frame was cut. Mounting and mechanical tests were carried out within 5 minutes after dissection of the wings, as we have previously shown that desiccation alters mechanical properties of cuticle very rapidly [14], [15].

To measure the strength of the membrane alone, samples were mounted as described above, however veins and surplus membrane material was cut away under a stereomicroscope, leaving only single membrane segments. Measurements on isolated membrane from within the remigium were not possible, as the wing cells were too small for our experimental setup.

The sample dimensions were measured after the test from the recorded video files. The membrane thickness of gold-coated wing samples was measured using a Tescan electron microscope (5 kV).

### Ethics Statement

All experiments were performed in the Trinity Centre for Bioengineering (TCD) in accordance with the Animals (Scientific Procedures) Act of 1986.

### Fracture Toughness, Stiffness and Strength

The fracture toughness of a material describes its resistance to crack propagation: fracture toughness testing involves measuring the stress required to propagate a pre-existing crack. To measure the fracture toughness of fresh and desiccated locust wings, we manually induced small cracks with various lengths at the edges of the mounted samples, parallel to the longitudinal veins.

All mechanical tests were performed using a standard tensile test machine (Zwick/Roell, 5N loadcell). Tension was always applied at a constantly increasing displacement of 0.1 mm/s until complete failure of the sample. Videos of each test were recorded at 25fps using a USB microscope at 20× magnification (Veho, United Kingdom) and initial crack length, sample dimension and the progress of crack propagation were measured after the test from the video files.

In the present samples, which were flat plates having equal length *L* and width *w*, with an edge crack *a* parallel to the clamped edges, and a stress σ at which the crack starts to propagate, the fracture toughness K_C_ for plane stress is defined as
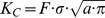
(1)


The geometrical correction factor *F* depends on the ratio a/w as follows [16]:
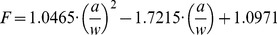
(2)


The initial fracture toughness K_C0_ was measured at the point where the crack started to propagate through the membrane (see Figure 2 a). We observed that the crack grew rapidly (causing a temporary drop in force) but stopped when it reached each cross vein, requiring an increase in stress before it could break through into the next cell. Using equations (1) and (2) we calculated the toughness when the crack broke through the first cross-vein (K_C1_), the second cross-vein (K_C2_) etc. (see Figure 2 b).

Ultimate tensile strength and stiffness of isolated membrane sections and complete wing sections were measured by applying tension until failure and measuring the strain from simultaneously recorded video files.

### Morphological Reconstruction of the Wing Cells

To automatically reconstruct the morphology of the wing cells, complete and undamaged hind wings from six female adult locusts were carefully cut off as close to the thorax as possible, spread out and fixed to a supporting sheet of paper and scanned using a standard office scanner at 1200 dpi resolution. After manual contrast and threshold adjustments for each image (using ImageJ), the vein structure and outline of the wing was reconstructed using Matlab. Small manual corrections were applied to the binary image to ensure an accurate and detailed representation of the cells where the automated algorithm fell short (for example ‘closing’ cell boundaries, see Figure S1).

A custom made Matlab script was then used to calculate the cell area, major axis length of each cell, and shortest distance to the outer wing edge from the centre of each wing cell. For statistical tests the results of each wing were grouped using the major-axis length with a bin size of 100 µm. Given a maximum resolution of 21.16 µm/pixel from the scanned images, wing cells with a major axis smaller than 100 µm were removed from the analysis.

### Statistics

Statistical tests [ANOVA, Fisher’s least significant difference (LSD) and paired *t-tests]* were performed where applicable using SPSS (Version 19, IBM, Armonk, NY, USA). If not stated otherwise, all values shown are means±s.d.; box-and-whisker plots show standard quartiles.

## Results

### Crack Propagation in the Locust Hind Wing

Fresh locust wings with a crack induced parallel to a longitudinal vein withstand a tensile force up to a certain limit, at which the crack starts to propagate through the wing. However, due to the pattern of stiff veins, this crack growth is not continuous yet follows a uniform pattern: i) cracks running towards a longitudinal vein are deflected once reaching the vein and continue to propagate parallel to the longitudinal vein. We have never observed a crack propagating through a longitudinal vein. ii) Cracks reaching a cross-vein are notably delayed or even stopped (see Video S1 and Figure S2 (video still)). When a crack reaches a cross vein, it arrests and, as the applied stress increases, the tip of the crack becomes blunt. The crack then either eventually propagates through the vein, or a secondary crack develops beyond the cross vein; the vein itself then fails, linking the secondary crack to the main crack (see Video S2).

If the initial crack was too small (less than approximately 700 µm) the specimen did not fail by propagation of the crack, but instead failed elsewhere, usually at the edge of the metal grips (see Video S3).

### Material Properties of the Membrane and the Wing

The fracture toughness of the membrane was measured by determining the stress at which a crack with a known length started to propagate through the material. Measurements of membrane thickness used for the calculation of stress values are summarized in Table 1.

**Table 1:** Average membrane thickness from three wing zones R, B and C (see Figure 1 a), measured from SEM cross sections.

Zone	Thickness (µm)
R	4.74±0.42 (n = 41)
B	3.05±0.71 (n = 60)
C	1.98±0.76 (n = 66)

Sample size shows number of measurements taken from 9 insects.

A comparison of the measurements taken from the wing zones R, B and C showed no significant differences in fracture toughness K_c0_ (F_2,16_ = 2.087, p>0.1, ANOVA, see Table 2 and Figure 2 c), allowing us to pool the measurements from all zones. Hence, the mean fracture toughness of the membrane itself, K_C0_, for all fresh segments was 1.04±0.25 MPa√m (N = 17).

**Table 2:** Summary of stiffness, strength and fracture toughness from fresh isolated wing membrane sections and complete wing sections (membrane plus veins) from three different regions of the hind wing (see Figure 1 a).

Zone	Membrane			Whole wing	
	Stiffness (GPa)	Strength (MPa)	K_C0_ (MPa√m)	Stiffness (MPa)	Strength (MPa)
R	–	–	1.15±0.25 (n = 5)	255.20±48.98 (n = 8)	25.52±4.89 (n = 8)
B	2.18±0.89* (n = 10)	56.26±9.82 (n = 10)	0.88±0.21 (n = 6)	233.93±55.95 (n = 6)	26.24±6.95 (n = 6)
C	1.45±0.28* (n = 8)	47.15±19.08 (n = 8)	1.11±0.25 (n = 6)	323.79±58.35 (n = 6)	32.37±5.83 (n = 6)
Mean	(1.86±0.77)	52.21±14.92 (n = 18)	1.04±0.25 (n = 17)	277.95±63.41 (n = 20)	27.79±6.34 (n = 20)

Significant differences are indicated.

* For details see Results.

To quantify the toughening effect of the veins, we measured the force required to overcome these barriers by calculating the fracture toughness K_CN_ at consecutive cross-veins (see Equation 1 and Figure 2 b). Our results show that the fracture toughness to overcome the first cross-vein K_C1_ was 1.57±0.38 MPa√m and thus significantly higher than the fracture toughness K_C0_ of the membrane alone (see Figure 2 d, t_9_ = −3.513, p<0.01, paired t-test). The resistance to fracture of the next wing cells K_C2_ - K_C4_ levels out at a mean value of 1.60±0.33 MPa√m.

We measured the ultimate tensile strength (UTS) and stiffness of isolated sections from fresh wing membrane on various different locations within the zones B and C (results are summarized in Table 2). Our results show that there was no significant difference in UTS between wing zone B or C (t_6.8_ = −1.131, p>0.05, two-tailed t-test). The mean ultimate tensile strength of the membrane from sections B and C together was 52.21±14.9 MPa (N = 18). The mean stiffness of fresh membrane sections from zone C was 1.45±0.28 GPa (N = 8) and from zone B 2.18±0.89 GPa (N = 10), which was significantly different (t_5.8_ = −2.200, p<0.05, two-tailed t-test).

The complete wing sections had a mean stiffness of 277.95±63.41 MPa, with no significant differences between the three zones (F_2,20_ = 3.357, p>0.05, ANOVA, see Table 2). The mean ultimate tensile strength of complete wing sections was 27.79±6.34 MPa, again with no significant differences between the three zones (F_2,20_ = 2.656, p>0.05, ANOVA, see Table 2).

### Vein Pattern Morphology of the Hind Wing

The morphological analysis of the hind wings showed that an average hind wing consists of 941±85 single wing cells. Together all cells cover an average membrane surface of 622.61±43.5 mm^2^, which is 78% of the overall mean wing area of 793.33±4.3 mm^2^ (membrane and veins, N = 6 wings from six adult female locusts, see Figure 3 a).

To estimate the maximum possible length a crack could travel within one cell, we calculated the “major axis length” for each single cell in every wing (Figure 3 a and b). The length of more than 99% of the wing cells was between 0.1 mm and 3.1 mm, with the most common group (7.88%) between 1.0 and 1.2 mm. The distribution of the cell’s length corresponds very well to a normal distribution around a mean value of 1.103 mm (σ = 544.16, a = 7.33, R = 0.98, N = 5553 cells from 6 wings).

The lengths of the cells increased with distance to the wing edge (sign. pos. correlation, ρ = 0.393, R^2^ = 0.154, p<0.001, see Figure 3 c). Smaller cells are mostly distributed around the perimeter of the wing with a notable accumulation of small cells within the remigium. Towards the centre of the wing the cells become longer.

## Discussion

The general function and morphology of insect wings, and in particular their vein structure, has been described in much detail [2], [3], [7]–[9], [13], [17], [18]. However, although the locust has been a biomechanical model organism for insect flight since the 1950s [8], [9], [19], there is very little experimental data available on the stiffness of locust wing material, and no data on its strength or fracture toughness [8], [10], [14].

In the following sections we will initially discuss the stiffness, strength and fracture toughness of the locust hind wing membrane alone. We will then show that the veins significantly improve the wing’s structural fracture toughness, and that in locusts the size and spatial distribution of the wing cells is very close to a biomechanical optimum.

### Material Properties of the Wing

Like all members of the Orthoptera group, locusts have two pairs of wings. The leathery forewings protect the underlying hind wings and assist in flight control [3], [20], whilst the membranous hind wings provide by far most of the aerodynamic lift and thrust during the downstroke [2], [3], [21]. It is thus seems likely that the locust hind wing membrane represents a “high-performance” adaptation to withstand high and repeated stresses during flight, in the same way that the hind legs of the locust are morphologically and mechanically adapted to perform long, energy efficient jumps [14], [22].

### Stiffness and Strength

In a previous study Smith *et al*. measured and discussed the stiffness of isolated membrane sections from *S. gregaria* locusts in great detail and at varying air humidity [8], [9]. The stiffness of the membrane found in the wing regions B (2.18±0.89 GPa) and C (1.45±0.28 GPa) measured in our study is in good agreement with values for comparable zones in locust wings by Smith *et al.*, which illustrates the validity of our experimental approach. In contrast to our stiffness measurements, our results however show no significant difference between the ultimate tensile strength of the wing membrane found in regions B (56.26±9.82 MPa) and C (47.15±19.08 MPa).

Our results partially support the idea of local anisotropy of insect wing-membrane stiffness, reported earlier by Smith et al [2], [8], [9]. However, these differences are relatively small and the values are based on average thickness measurements of the membrane (see Table 1). As all our other measurements of stiffness and strength from both membrane and whole wings showed no significant differences (see Table 2), we believe that for basic modelling it is a justified approach to simplify the wing and the wing’s membrane using mean values for stiffness and strength. Future studies using more sensitive experimental techniques should investigate whether the properties of the wing membrane might vary radially.

Interestingly, although histologically very different, the wing membrane’s mean strength (52.21±14.92 MPa) and mean stiffness (1.86±0.77 GPa) is relatively similar to the strength and stiffness of fresh locust tibia cuticle (72.05±30.5 MPa; 3.05±0.6 GPa [14]), the stiffness of dragonfly wings (2.85 GPa [2], [3], [11], [23]) and beetle wings (1.63 to 3.88 GPa [9], [11], [13], [24]). These similarities indicate that although insect cuticle is known for the versatility of its mechanical properties [25], the actual range of these properties might be considerably smaller. At least within one species, some exoskeleton body parts, such as wings and legs, might show a greater mechanical similarity than previously thought. If this is the case, this could significantly facilitate future biomechanical simulations and modeling. Additional comparative studies should investigate whether similar mechanical properties can be found in other body parts of *S. gregaria* locusts or even other insect species.

The comparison of the membrane and complete wing properties showed that the presence of the cross veins decreased the wing’s effective strength by almost 46% and the effective stiffness by 85% (see Table 2).

### Fracture Toughness and Work of Fracture

Our results show that with a mean value of 1.04 MPa√m the fracture toughness of fresh locust wing membrane is surprisingly low compared to other parts of the insect body. In a recent study we showed that the cuticle of fresh hind leg tibia of *S. gregaria* locusts, the same species, had a mean fracture toughness of 4.12 MPa√m, which is more than four times higher than the fracture toughness of the fresh wing membrane. With a fracture toughness of 1.04 MPa√m and a mean stiffness of 1.86 GPa the fresh locust wing membrane shows a work of fracture G_C_ of only 0.58 kJ/m^2^, which is also very low compared to an more than 10 times higher value of 5.56 kJ/m^2^ for the fresh hind leg tibia of the locusts [14]. Interestingly, the work of fracture of fresh locust wing membrane is in much better agreement with 0.68 kJ/m^2^ of *dry* locust legs [14]. Smith *et al.* have already shown that the mechanical properties of the wing membrane are only very little affected by humidity [8], [9]. It thus might be the case that locusts have adapted their wing morphology to the use of “dry” cuticle. Given that locusts are primarily found in dry deserts, this approach seems more advantageous than either investing in evaporation protection (increasing wing weight) or constantly losing water whilst keeping wings humidified.

### Effect of Cross-veins on Fracture Toughness

The vein pattern of insect wings shows a great variety, yet is known to be species specific and has been used in the past for taxonomic identification purposes [8], [10], [26]. Besides their importance for wing performance during flight, it has been suggested that in insect wings these veins might also act as defect “barriers” by preventing cracks from growing [2], [3], [8]. However, until now there has been no experimental evidence for this hypothesis.

Our results show that the venation of the locust hind wings not only qualitatively but also quantitatively follows a remarkably uniform pattern. Local distribution and also the total number and the size of cells were almost identical in each hind wing.

As our fracture toughness results show, the wing veins are effective crack barriers. The presence of cross veins significantly increases the effective structural fracture toughness of the wing by 50% from 1.04 to 1.57 MPa√m (see Figure 2 d). Many materials increase their toughness by making use of barriers to crack propagation: examples are grain boundaries in metals, fibres in composite materials and osteon cement lines in bone [4]. We have previously developed a theoretical model to describe the effect of these barriers and of the crack growth jumping mechanism which they induce [27].

At first glance it thus seems beneficial to have many cross veins forming small wing cells, capturing and “blunting” membrane defects as soon as possible. Similar to the watertight compartments of a ship, a defect in the membrane might be effectively captured and blunted by the surrounding vein material. However, a high number of veins would not only reduce the flexibility of the wing [28], but also increase the overall weight considerably [17]. Given a biomechanical relation of veins and fracture toughness, the size of the wing cells could be optimized in respect to high fracture toughness with a minimum of mass.

### Toughest Wing Cell Size

An optimal cell size of a grid-like structure such as the wing can be predicted using the “critical crack length” of the membrane, which is determined by the material’s fracture toughness and the stress applied. At a given stress, any crack smaller than the critical length would have no structural effect. As a consequence, the largest possible cell size that prevents cracks from self propagating corresponds to this critical crack length. If a crack is contained within this cell, it cannot reach a critical length to self propagate through the rest of the wing. Any cell *bigger* than this critical crack length would allow the initial crack to start growing. However, any cell *smaller* than this critical crack length would be a “waste” of vein material. Thus the important question in respect to the optimization of insect wing cell size now is: what is the critical crack length of the wing membrane alone?

To calculate the critical crack length with a known mean fracture toughness of 1.57 MPa√m, we need to estimate the maximum stress a locust wing experiences (see Equation 1). Obviously, during natural flight the hind wing of an insect is subject to various, turbulent stresses. So far there is no data available on the stress acting on a locust wing during untethered flight [20]. However, we believe it is justified to assume that during normal flight, the local stress on the wing should never exceed the ultimate tensile strength (UTS) of the membrane, which is presumably its weakest component. Any local stress higher than the membrane’s UTS would result in immediate failure of the wing cell. We therefore take a strength of 37 MPa as the “worst case” stress the hind wing membrane should experience (mean UTS 52.21 MPa −14.92 MPa s.d.).

Following equation 1, this results in a critical crack length for an edge crack of 566 µm. For a central crack, induced for example in the middle of a membrane field, this value doubles to 1132 µm. Consequently, any crack *smaller* than 1132 µm will *not* self propagate through the locust wing membrane up to a stress as high as the ultimate tensile strength of the material. A wing-cell length of 1132 µm thus represents an upper boundary to protect the locust wing from centre cracks growing. An ‘optimal’ wing cell should have a diameter of around 1132 µm. Is this the case in locust wings?

Our results show that the distribution of the wing cell size in locust wings corresponds very well to this prediction, with the most common wing-cell “class” being between 1000 and 1100 µm (see Figure 3 b). Interestingly, wing cells smaller than the critical crack length contribute to only 19.44% of the wing’s overall membrane area. However, as stated before, this is a worst-case scenario approach for a local stress. If we look at complete wing’s UTS of 27.79 MPa (see Table 2), the critical crack length for a centre crack increases to 2036 µm (1018 µm for an edge crack). More than 91% of all wing cells are covered within this range, adding up to 77.8% of the membrane area and 60.7% of the overall wing area.

Interestingly, our results also show that the wing cell size is not evenly distributed within the wing area. Some regions show an accumulation of smaller cells, in particular the remigium (see Figure 3 a). The larger cells however are mostly located in the centre of the wing. Our results also show a significant positive correlation of wing cell size with distance to the edge. One possible explanation might be that wing cells at the edge of the wing might be susceptible to physical damage and tear and experience higher stresses during flight. Their smaller size increases the structural resistance to crack propagation. In addition, losing one single small edge-cell at the edge would not notably affect the wing’s total area. Wing cells in the middle of the wing on the other hand might be less likely to get damaged and therefore would allow for bigger areas. Lower stresses experienced by the membrane in the middle of the wing also allow for a larger critical crack length, therefore larger cells.

However, the optimization of fracture toughness is obviously not the only boundary condition in the biomechanical “design” of the locust wing venation pattern. Previous studies on locusts, fly and dragonfly-wings have shown the importance of the wing vein pattern in the distribution of stresses during flight [13], [29], [30]. Other constraints such as flexibility, rigidity and corrugation of the wing also play a very important role and lead to the morphological derivations from the “optimal” fracture toughness design.

### A Tool for Comparative Biomechanics

Preliminary analyses using previously published photographs of insect wings indicate that a similar distribution of wing cell size might be found in the wings of recent Odonata (dragonflies) and even in fossilized dragonfly wings (see Figure S1). Future studies, comparing the venation pattern of different insect species in respect to their wing cell size and correlating these results with mechanical tests are planned investigate this observation in more detail. If the correlation between shape and properties found in this study is shown to be valid in other insect species, it might be a helpful tool to study the mechanical properties of endangered or even extinct insects’ cuticle.

### Technical and Biomimetic Applications

Engineers designing micro-air-vehicles (MAV) are more and more inspired by the amazing flight performance of insects. Lightweight flapping robots are now capable of untethered stable flight using membranous artificial wings for several minutes [31]–[33]. However, the durability and performance of artificial and biological flapping wings is greatly reduced by mechanical wear through clapping [34]. As continuous flight times keep increasing more and more, due to better batteries and efficient actuators, defects and fatigue in artificial wings might significantly limit the life expectancy of a flapping MAV. Incorporating a material-specific biomimetic cell-like structure into future designs of membranous wings could increase the wings’ durability, yet keep additional weight at a minimum.

## Supporting Information

Figure S1 **Comparison of locust wing patterns to dragonfly wings.** (a) Size and distribution of wing cells in the hind wings of the recent dragonfly *Sympetrum vulgatum* (adapted from [35]) and the extinct *Protolindenia viohli* (fossil imprint from the upper Jurassic, adapted from [36]). The wing venation pattern of dragonflies barely changed within the last hundreds of millions of years [35], [37]. (b) Both wings show a distribution of wing cells very similar to that of *S. gregaria* (same data as in Figure 3 b), with the *P. viohli* wing showing a higher number of smaller wing cells, in particular at the edge of the wing. The pterostigmata have been removed from the analysis.
(TIF)

Figure S2 **(video still) Propagation of a crack through a hind wing of** ***S. gregaria*** **under tension.** At a certain stress the initial crack starts propagating through the membrane (K_C0_). When the crack hits a cross vein (subfigures b, c and d), the crack is delayed, which increases the fracture toughness of the wing (K_C1_). Numbers indicate frame number.
(TIF)

Video S1 **(online only) Video of a manually induced crack propagating through a hind wing of** ***S. gregaria*** **under tension.** At a certain tensile stress the initial crack starts propagating through the membrane (K_C0_). Hitting cross veins notably inhibits the cracks propagation, which increases the fracture toughness of the wing (K_C1_).
(MP4)

Video S2 **(online only) The initial crack is stopped by a cross vein.** A secondary crack develops beyond the cross vein; the vein itself then fails, linking the secondary crack to the main crack.
(MP4)

Video S3 **(online only) Edge cracks with an initial length less than approximately 700 µm (left side) do not propagate through the wing, which leads to tensile failure at the clamping.**
(MP4)

## References

[1] Lovejoy NR, Mullen SP, Sword GA, Chapman RF, Harrison RG (2006) Ancient trans-Atlantic flight explains locust biogeography: molecular phylogenetics of Schistocerca. Proc R Soc Lond B 273: 767–774 doi: 10.1098/rspb.2005.3381. 10.1098/rspb.2005.3381 PMC1560218 16618668

[2] Wootton RJ (1992) Functional Morphology of Insect Wings. Annu Rev Entomol 37: 113–140 doi: 10.1146/annurev.en.37.010192.000553.

[3] Wootton RJ, Evans K, Herbert RC, Smith C (2000) The hind wing of the desert locust *(Schistocerca gregaria* Forskal) I. Functional morphology and mode of operation. J Exp Biol 203: 2921–2931. 10976029 10.1242/jeb.203.19.2921

[4] Taylor D, Hazenberg JG, Lee TC (2007) Living with cracks: damage and repair in human bone. Nat Mater 6: 263–268 doi: 10.1038/nmat1866. 17401419 10.1038/nmat1866

[5] Bloch R (1941) Wound healing in higher plants. Bot Rev 7: 110–146 doi: 10.1007/BF02872446.

[6] Martin P (1997) Wound healing - Aiming for perfect skin regeneration. Science 276: 75–81 doi: 10.1126/science.276.5309.75. 9082989 10.1126/science.276.5309.75

[7] Lai-Fook J (1968) The Fine Structure of Wound Repair in an Insect (*Rhodnius prolixus*). J Morphol 124: 37–77. 4297319 10.1002/jmor.1051240104

[8] Smith C, Herbert RC, Wootton RJ, Evans K (2000) The hind wing of the desert locust (Schistocerca gregaria Forskal) II. Mechanical properties and functioning of the membrane. J Exp Biol 203: 2933–2943. 10976030 10.1242/jeb.203.19.2933

[9] Herbert RC, Young P, Smith C (2000) The hind wing of the desert locust (*Schistocerca gregaria* Forskal). III. A finite element analysis of a deployable structure. J Exp Biol 203: 2945–2955. 10976031 10.1242/jeb.203.19.2945

[10] Hamilton KGA (1992) The Insect Wing, Part III. Venation of the Orders. J Kansas Entomol Soc 45: 145–162.

[11] Wootton RJ (1995) Geometry and Mechanics of Insect Hindwing Fans: A Modelling Approach. Proc R Soc Lond B 262: 181–187 doi: 10.1098/rspb.1995.0194.

[12] Marcus JM (2001) The development and evolution of crossveins in insect wings. J Anat 199: 211–216 doi: 10.1046/j.1469-7580.2001.19910211.x. 11523825 10.1046/j.1469-7580.2001.19910211.x PMC1594997

[13] Ennos AR (1989) Comparative functional morphology of the wings of Diptera. Zool J Linnean Soc 96: 27–47 doi: 10.1111/j.1096-3642.1989.tb01820.x.

[14] Dirks J-H, Taylor D (2012) Fracture toughness of locust cuticle. J Exp Biol 215: 1502–1508 doi: 10.1242/jeb.068221. 22496286 10.1242/jeb.068221

[15] Dirks J-H, Dürr V (2011) Biomechanics of the stick insect antenna: Damping properties and structural correlates of the cuticle. J Mech Behav Biomed 4: 2031–2042 doi: 10.1016/j.jmbbm.2011.07.002. 10.1016/j.jmbbm.2011.07.002 22098903

[16] Torvik PJ (1979) On the Determination of Stresses, Displacements, and Stress-Intensity Factors in Edge-Cracked Sheets With Mixed Boundary Conditions. J Appl Mech 46: 611 doi: 10.1115/1.3424615.

[17] Dudley R (2000) The biomechanics of insect flight. Chichester: Princeton University Press.

[18] Combes S, Daniel T (2003) Flexural stiffness in insect wings I. Scaling and the influence of wing venation. J Exp Biol 206: 2979–2987 doi: 10.1242/jeb.00523. 12878666 10.1242/jeb.00523

[19] Weis-Fogh T, Jensen M (1956) Biology and Physics of Locust Flight. I. Basic Principles in Insect Flight. A Critical Review. Philos Trans R Soc Lond B Biol Sci. doi: 10.1098/rstb.1956.0007.

[20] Walker SM, Thomas ALR, Taylor GK (2009) Deformable wing kinematics in the desert locust: how and why do camber, twist and topography vary through the stroke? J R Soc Interface 6: 735–747 doi: 10.1098/rsif.2008.0435. 19091683 10.1098/rsif.2008.0435 PMC2841574

[21] Weis-Fogh T (1956) Biology and Physics of Locust Flight. II. Flight Performance of the Desert Locust. Philos Trans R Soc Lond B Biol Sci 239: 459–510 doi: 10.1098/rstb.1956.0008.

[22] Sutton GP (2011) An exemplar of energy accounting: The energetics of the locust jump. J Exp Biol 214: 2997–2998 doi: 10.1242/jeb.056663. 21865510 10.1242/jeb.056663

[23] Song F, Xiao KW, Bai K, Bai YL (2007) Microstructure and nanomechanical properties of the wing membrane of dragonfly. Mater Sci Eng, A 457: 254–260 doi: 10.1016/j.msea.2007.01.136.

[24] Ha NS, Jin TL, Goo NS, Park HC (2011) Anisotropy and non-homogeneity of an Allomyrina Dichotomabeetle hind wing membrane. Bioinspir Biomim 6: 046003 doi: 10.1088/1748-3182/6/4/046003. 21992989 10.1088/1748-3182/6/4/046003

[25] Vincent JFV, Wegst UGK (2004) Design and mechanical properties of insect cuticle. Arth Struct & Dev 33: 187–199 doi: 10.1016/j.asd.2004.05.006. 10.1016/j.asd.2004.05.006 18089034

[26] Tofilski A (2004) DrawWing, a program for numerical description of insect wings. J Insect Sci 4: 17. 15861233 10.1093/jis/4.1.17 PMC528877

[27] Taylor D (2007) The Theory of Critical Distances. 1st ed. London: Elsevier Science.

[28] Combes S, Daniel T (2003) Flexural stiffness in insect wings II. Spatial distribution and dynamic wing bending. J Exp Biol 206: 2989–2997 doi: 10.1242/jeb.00524. 12878667 10.1242/jeb.00524

[29] Meng XG, Xu L, Sun M (2011) Aerodynamic effects of corrugation in flapping insect wings in hovering flight. J Exp Biol 214: 432–444 doi: 10.1242/jeb.046375. 21228202 10.1242/jeb.046375

[30] Kesel AB (2000) Aerodynamic characteristics of dragonfly wing sections compared with technical aerofoils. J Exp Biol 203: 3125–3135. 11003823 10.1242/jeb.203.20.3125

[31] Khan ZA, Agrawal SK (2011) Study of Biologically Inspired Flapping Mechanism for Micro Air Vehicles. Aiaa J 49: 1354–1365 doi: 10.2514/1.J050447.

[32] Richter C, Lipson H (2011) Untethered hovering flapping flight of a 3D-printed mechanical insect. Artif Life 17: 73–86 doi: 10.1162/artl_a_00020. 21370958 10.1162/artl_a_00020

[33] Park JH, Yoon K-J (2008) Designing a Biomimetic Ornithopter Capable of Sustained and Controlled Flight. J Bionic Eng 5: 39–47 doi: 10.1016/S1672-6529(08)60005-0.

[34] Ellington CP (1999) The novel aerodynamics of insect flight: applications to micro-air vehicles. J Exp Biol 202: 3439–3448. 10562527 10.1242/jeb.202.23.3439

[35] Jongerius SR, Lentink D (2010) Structural Analysis of a Dragonfly Wing. Exp Mech 50: 1323–1334 doi: 10.1007/s11340-010-9411-x.

[36] Nel A, Bechly G, Martínez-Delclòs X (2001) A new fossil dragonfly from the Upper Jurassic of Germany (Odonata: Anisoptera: Protolindeniidae). Rev Fr Entomol 23: 257–261.

[37] Wootton RJ (1998) Smart Engineering in the Mid-Carboniferous: How Well Could Palaeozoic Dragonflies Fly? Science 282: 749–751 doi:1 0.1126/science.282.5389.749. 9784134 10.1126/science.282.5389.749

