# Supplementary figures and images for: Veins Improve Fracture Toughness of Insect Wings
Source: PLoS One. 2012 Aug 22;7(8):e43411. doi: 10.1371/journal.pone.0043411 (PMC3425546; doi:10.1371/journal.pone.0043411)

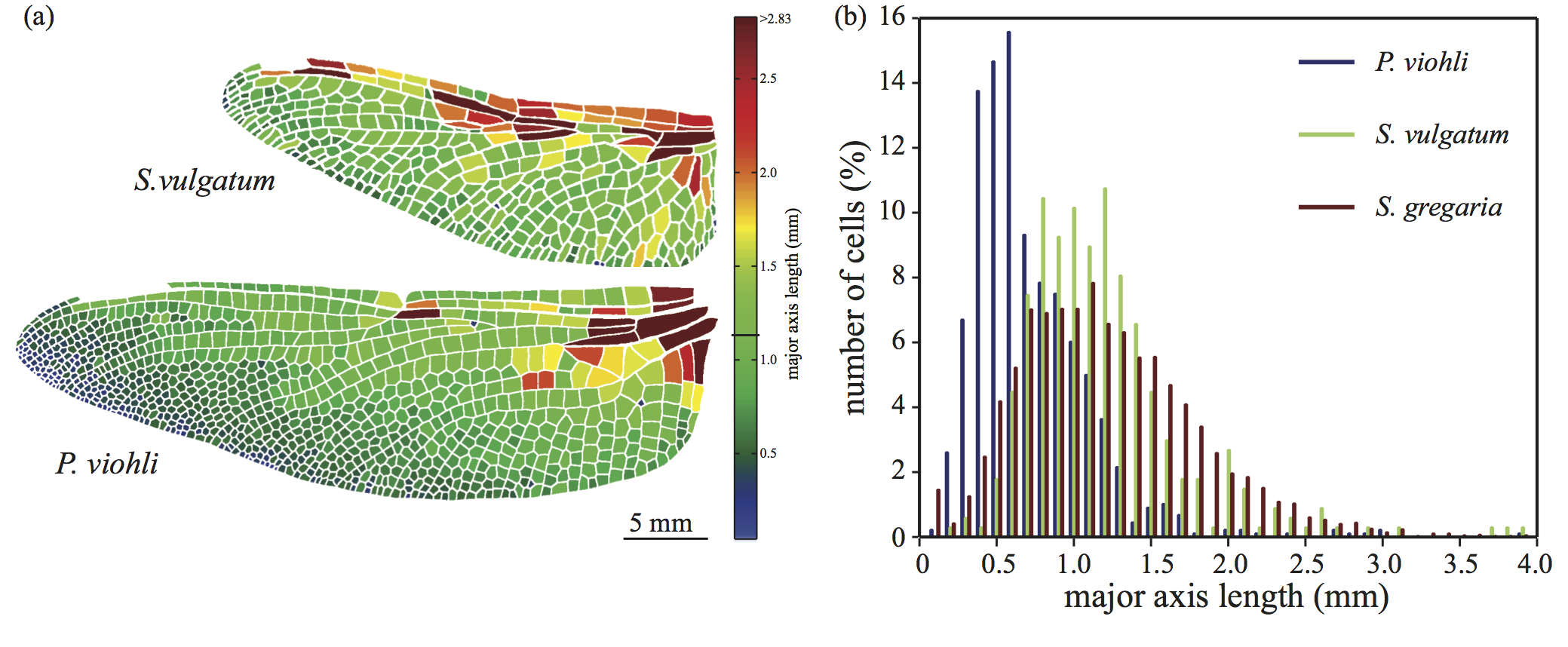

Supplement: Figure S1 — Comparison of locust wing patterns to dragonfly wings. (a) Size and distribution of wing cells in the hind wings of the recent dragonfly Sympetrum vulgatum (adapted from [35]) and the extinct Protolindenia viohli (fossil imprint from the upper Jurassic, adapted from [36]). The wing venation pattern of dragonflies barely changed within the last hundreds of millions of years [35], [37]. (b) Both wings show a distribution of wing cells very similar to that of S. gregaria (same data as in Figure 3 b), with the P. viohli wing showing a higher number of smaller wing cells, in particular at the edge of the wing. The pterostigmata have been removed from the analysis. (TIF) [file pone.0043411.s001.tif]

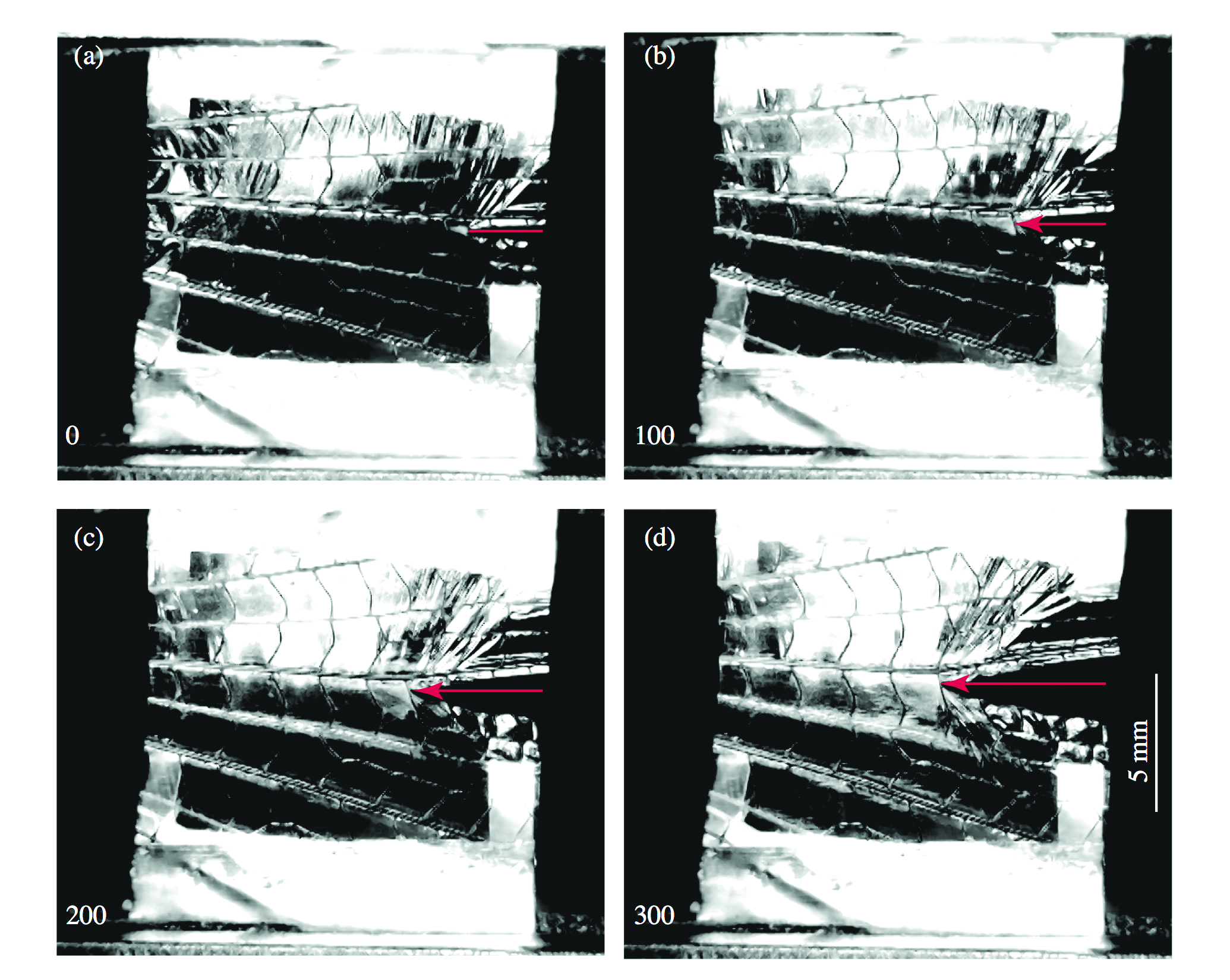

Supplement: Figure S2 — (video still) Propagation of a crack through a hind wing of S. gregaria under tension. At a certain stress the initial crack starts propagating through the membrane (KC0). When the crack hits a cross vein (subfigures b, c and d), the crack is delayed, which increases the fracture toughness of the wing (KC1). Numbers indicate frame number. (TIF) [file pone.0043411.s002.tif]
